# A novel *APOL1* membrane-addressing domain mutation (*p.T272I*) in Chinese twins with FSGS: implications for podocyte injury and ion channel disruption

**DOI:** 10.1080/0886022X.2025.2553384

**Published:** 2025-09-15

**Authors:** Ting Peng, Dan Chang, Tingyu Chen, Sipei Chen, Li Wang, Guisen Li

**Affiliations:** ^a^ Department of Nephrology and Nephrology Institute, Sichuan Provincial People’s Hospital, School of Medicine, University of Electronic Science and Technology of China, Chengdu, China; ^b^ Anti-infective Agent Creation Engineering Research Centre of Sichuan Province, Sichuan Industrial Institute of Antibiotics, School of Pharmacy, Chengdu University, Chengdu, China; ^c^ Department of Nephrology, Chengdu First People’s Hospital, Chengdu, China

**Keywords:** Apolipoprotein L1, focal segmental glomerulosclerosis, podocyte, cytoskeleton, chloride channel

## Abstract

Apolipoprotein L1 (APOL1) risk variants are strongly associated with kidney diseases, including focal segmental glomerulosclerosis (FSGS), though known mutations (G1/G2) are primarily observed in African populations. This study reports a novel APOL1 mutation (p.T272I) identified in a pair of Chinese twins with FSGS, expanding the genetic spectrum of APOL1-related nephropathies. Whole exome sequencing detected the APOL1 mutation in the twins and excluded parental inheritance. Functional studies demonstrated that transfection of mutant APOL1 into human podocytes and HEK293T cells led to cytoplasmic lysis, disrupted F-actin organization, and reduced expression of nephrin and synaptopodin. The variant also induced mitochondrial dysfunction, increased the LC3-II/I ratio, activated p38 MAPK signaling, and altered chloride channel activity. Structural modeling *via* AlphaFold2 suggested conformational disturbance within the membrane-addressing domain. These findings reveal the first de novo APOL1 mutation in the Chinese population, implicating podocyte injury through cytoskeletal collapse, mitochondrial damage, altered autophagic markers, and ion channel dysfunction in FSGS pathogenesis. This study expands the spectrum of APOL1-related variants beyond G1/G2 and highlights underlying mechanisms for potential therapeutic targeting.

## Graphical Abstract

**Figure:**
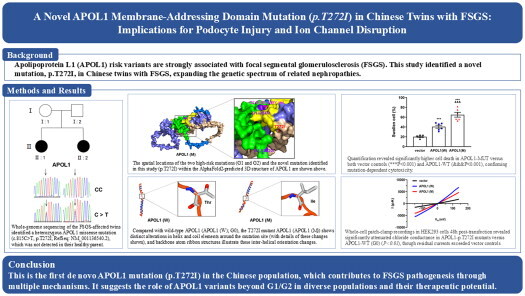

## Introduction

Focal segmental glomerulosclerosis (FSGS) constitutes a pathological subtype of resistant nephrotic syndrome (NS) among children and young patients. It is characterized by diffuse fusion or effacement of podocyte foot processes [1,2]. Over the past two to three decades, the incidence of patients progressing to end-stage renal disease (ESRD) due to secondary FSGS has surged by nearly 11-fold [3]. Genetic mutations on an individual Mendelian basis or the augmented influence of environmental factors can amplify the predisposition to FSGS. A study reports that approximately 8% of FSGS patients have associations with genetic mutations and familial genetic backgrounds [4].

In recent decades, a succession of genes encoding podocyte-related molecules has been correlated with FSGS, including *NPHS1*, *NPHS2*, *ACTN4*, *TRPC6*, *INF2* and *APOL1* [5–8]. Among these, Apolipoprotein L1 (*APOL1*) has emerged as a gene of particular interest due to its central role in *APOL1*-associated nephropathies, a group of disorders where FSGS represents a prominent clinical manifestation [9,10]. Thus, investigating FSGS from a genetic standpoint, with a specific focus on *APOL1* and its associated nephropathies, has become pivotal for elucidating the disease’s pathogenesis.

*APOL1* serves as a circulating innate immunity protein and assumes a crucial role in the lysis of trypanosomes, which are parasites that cause sleeping sickness in infected individuals [11]. In the kidney, *APOL1* finds expression in both podocytes and proximal tubules [12]. Extensive research has demonstrated that two specific risk variants of *APOL1* (*G1* and *G2*) significantly contribute to kidney disease progression, particularly within the context of *APOL1*-associated nephropathies [13–15]. This gain-of-function mutation in *APOL1* triggers podocyte toxicity through endoplasmic reticulum stress, autophagy, and disruptions in ion flow, leading to the development of associated nephropathies such as FSGS [9,16–18]. Notably, *APOL1*-mediated ion transport involves both anionic and cationic fluxes, which play distinct roles in renal pathophysiology [19–21]. Chloride ions, critical for maintaining intracellular osmotic balance and membrane potential in podocytes, contribute to cellular volume regulation—an essential process for preserving the integrity of the glomerular filtration barrier [20]. Cations such as potassium, sodium, and calcium, on the other hand, are pivotal for intracellular signaling, cytoskeletal dynamics, and mitochondrial function in podocytes [19,22–24]. Disruptions in their transport can impair podocyte viability and function. Prior studies have highlighted *APOL1*’s dual capacity to transport these ions under varying pH conditions, with risk variants like *G1* and *G2* predominantly altering cation flux, while wild-type *APOL1* (G0) exhibits chloride-selective conductance under certain physiological context [24,25].

The association between *APOL1* genetic variants and kidney disease in African Americans (AAs) was first established by landmark studies from Genovese G et al. and Tzur S et al. which identified these variants as critical drivers of renal pathology in this population [26,27]. Subsequent research has further elaborated that *APOL1* mutations inherited in a recessive manner confer a striking 17-fold increased risk of progression from FSGS to end-stage renal disease (ESRD) among AAs, with podocyte injury recognized as a key pathogenic mechanism [28]. Notably, the primary pathogenic variants identified—*G1* and *G2*—exhibit a strong population specificity, being predominantly associated with the AA cohort [26,27,29,30]. In the present study, we have successfully identified a novel *APOL1* mutation (*c.C815T*, *p.T272I*; RefSeq: NM_001136540.2) in a twin patient with FSGS through gene sequencing techniques. Our primary investigation was centered on understanding the impact of this newly identified mutation on podocytes and elucidating its connection to the pathogenesis of FSGS.

## Methods

### Subjects

Clinical and laboratory data were meticulously gathered from medical records as well as computer-based data repositories at the Sichuan Provincial People’s Hospital. All kidney biopsy specimens were assigned a histopathologic diagnosis by a clinical pathologist. This study was approved (Approval Number: 2018283) by the Institutional Review Boards of both the Sichuan Academy of Medical Sciences and the Sichuan Provincial People’s Hospital. Written informed consents were obtained from the two subjects prior to studies.

### Whole exome sequencing and mutant plasmid construction

Genomic DNA was extracted from the peripheral blood of the two cases and their parents. Genetic analysis was performed by massively parallel sequencing in the genetics laboratories of MyGenostics biotechnology companies in China. The *APOL1* genotypes of renal disease risk variants in the deidentified biopsy samples of individuals with FSGS were determined using DNA extracted from peripheral blood cells. Whole exome sequencing was performed on the proband using the standard protocol.

pCMV6-entry vector encoding the transcript variant 3 of *APOL1* cDNA (RefSeqORF: 1197 bp) with C-terminal MYC and DDK tags was purchased from OriGene (*RG226566*). The *APOL1* new variants were generated using the QuikChange II Site-Directed Mutagenesis kit (Agilent Technologies, Cat. 200524), Forward primer: *5′-CCTTAGCTGGCAATATTTACCAACTCACACG-3′*, Reverse primer: *5′-CGTGTGAGTTGGTAAATATTGCCAGCTAAGG-3′*. Sequencing verifies the correctness of the mutation site.

### Histopathology assay

The tissues were fixed in 10% formalin solution, embedded in paraffin, and sectioned to 4 µm on standard microscopy slides. The sections were then heated overnight at 40 °C and then at 62 °C for 1 h. Paraffin was cleared with xylenes, and the section was rehydrated in a graded ethanol series and immersed in ddH_2_O. The sections were then stained with Periodic Acid-Schiff stain and Periodic Acid-Methenamine Sliver stain. For immunohistochemistry (IHC), slides were transferred to a pressure boiler containing boiling antigen retrieval solution (10 mM sodium citrate-hydrochloric acid buffer solution, pH = 6.0), sealed and heated for an additional 3 min. Slides were cooled to 25 °C and rinsed in phosphate buffer saline (PBS, pH = 7.4). Endogenous peroxidase activity was blocked for 15 min at room temperature with 3% H_2_O_2_ in methanol, then washed in PBS and blocked for 1 h at room temperature in 5% serum in PBS. Sections were then incubated overnight at 4 °C in 1% serum, PBS with rabbit anti-*APOL1* primary antibody (Abcam, Cat. ab252218) at 1:500 [31,32]. After washing with PBS, the slices were incubated with the appropriate secondary antibody for 30 min at 37 ◦C. Peroxidase activity was revealed using 3,3-diaminobenzidine (DAB, Solarbio, DA1010) and counterstained with hematoxylin. Then, the slides were rinsed in tap H20, dehydrated in a graded ethanol series then xylenes. Images were captured using an Olympus Bx51 microscope, and images were captured with an Olympus DP71 camera with DP controller software (Olympus).

### APOL1 protein structure modeling

Download the source code and install it to the server according to the AlphaFold2 code that the Deepmind team has posted on https://gitcode.net/mirrors/deepmind/alphafold. Enter the *APOL1* amino acid sequence of the T272I mutation for 3-dimensional structure modeling, and download the .pdb file. The .pdb file of wild-type *APOL1* protein (AF-O14791-F1) was downloaded from https://alphafold.ebi.ac.uk/entry/O14791. Differences in the structure of mutant and wild-type proteins were performed on AutoDock Vina and AutoDock Tools, and the results obtained were opened with Pymol and LigPlus for preview, analysis, and graphing. Input the T272I mutant and wild-type amino acid sequences derived from *APOL1* isoform b (NM_145343.3, which has a longer N-terminus compared to isoform a) separately to the website of http://bioinf.cs.ucl.ac.uk/psipred/ for online prediction protein secondary structure.

### Cell culture

The conditionally human immortalized podocyte cell line, kindly provided by Nanjing Military Region General Hospital, was cultured as described [33]. The cells were cultured in RPMI 1640 medium (Hyclon, Cat. SH30809.01) supplemented with 10% FBS (Gibco^™^ Fetal Bovine Serum, Qualified, Cat. 26140095), 1% penicillin-streptomycin, 1% Insulin-Transferrin-Selenium-A (Gibco, Cat. 41400045), and 40 U/ml γ-IFN under permissive conditions at 33 °C and 5% CO_2_. For differentiation, cells were transferred to non-permissive conditions at 37 °C, removing ITS and γ-IFN compared to the preceding medium. Cells were harvested after 7 to 14 days of differentiation. HEK293T cells were grown under 5% CO_2_ at 37 °C in Dulbecco’s modified Eagle’s (Hyclon, Cat. SH30022.01) supplemented with 10% FBS and 1% PS.

### Transfection of APOL1 plasmid

The differentiated podocytes were seeded in six-well plates (1 × 10^5^ cells/well), starved for 2 h with serum-free medium before transfection, equal amounts *APOL1* plasmid negative control mixed with Lipofectamine 3000 (Invitrogen, Cat. L3000015) and Opti-MEM (Gibco, Cat. 31985062) according to the manufacturer’s instructions. The mixture was added to the cells and replaced with a complete medium after 6 h. A follow-up experiment was performed 48 h later.

### Protein extraction and Western blotting

Total cellular protein was isolated by lysing the cells with RIPA buffer (Beyotime, Cat. P0013D) supplemented with protease and phosphatase inhibitors, and protein was quantified using a bicinchoninic acid–based (BCA) assay (Pierce, Cat. 23227). Equal quantities of total proteins were separated by SDS-PAGE and transferred to a PVDF membrane. After transfer, membranes were blocked in 5% nonfat dry milk at room temperature for at least 1 h. Anti-*APOL1* (Abcam, Cat. ab108315) [25], anti-Nephrin (Abcam, Cat. ab58968), anti-Synaptopodin (Abcam, Cat. ab224491), anti-LC3(CST, Cat. 3868), anti-*p38* MAPK (CST, Cat. 8690) and anti-p*p38* MAPK (CST, Cat. 4511) primary antibodies diluted at 1:1000 was incubated with membranes overnight at 4 °C. Membranes were washed three times with TBST, and incubated with anti-rabbit horseradish peroxidase-conjugated secondary antibody (Proteintech, Cat. SA00001-2, 1:2000) at room temperature for 1 h. The membrane was further developed with the ECL Plus Western blotting detection system (GE Healthcare). To control the equal amount of protein loading all detected proteins were densitometric normalized to GAPDH (Proteintech, Cat. 10494-1-AP).

### Immunofluorescence staining

Cells were fixed in 3.7% paraformaldehyde for 10 min at room temperature, washed with PBS 3 times, and permeabilized using 0.1% Triton X-100 in PBS for 5 min, followed by blocking with 1% BSA for 30 min. Filamentous-actin (F-actin) was visualized using 1 U Alexa Fluor 568 Phalloidin (Invitrogen, Cat. A12380), Mitochondrial marked by 200 nM Mito-Tracker Red CMXRos (Beyotime, Cat. C1049), one glass slide with 200 μL above dilution incubated overnight at 4 °C. After extensive washes, 200 μL DAPI was utilized as a nuclear counterstain and incubated for 30 min at room temperature. Images were acquired by immunofluorescence microscopy (Leica TCS SP8, Germany).

### Patch clamp

Cells were seeded in a 6-well plate and subjected to transfect plasmid for 48 h. Experiments were conducted in three independent replicates (*N* = 3), with each replicate including three experimental groups (Vector control, *APOL1*-WT, *APOL1*-T272I). For each group, 3–5 cells meeting selection criteria were analyzed: (1) Intact morphology (no membrane damage/blebbing); (2) Stable gigaseal (>1 GΩ) maintained ≥2 min; (3) Resting potential −60 to −80 mV (fluctuation <5 mV); (4) No current leakage at −70 mV holding potential [25,34]. Mechanical manipulation of pipettes was monitored with a Nikon inverted microscope when starting the detection. A capacitive current transient was induced by a hyperpolarizing square pulse of voltage from −150 to −180 mV and recorded at 10 KHz to generate current-voltage (I-V) relations. All recordings were made at room temperature (approximately 22 °C). Membrane capacitance was calculated offline by integrating the area of the capacitive transient at the onset of the pulse, then dividing the integrated by the amplitude of the pulse (−10 mV). Data were obtained using a borosilicate electrode (Sutter Instruments). Calculation of the integration was made with the clamp fit module of pClamp 10.0 software (Molecular Devices). Pippette solution contained 10 mM NaCl, 125 mM CsCl, 6.2 mM MgCl_2_, 10 mM HEPES, and 10 mM EGTA, pH 7.2. The normal pipette solution was composed of 150 mM NaCl, 5.4 mM CsCl, 0.8 mM MgCl_2_, 5.4 mM CaCl2, and 10 mM HEPES, pH 7.4.

### Statistical analysis

Data are expressed as means ± SEM and analyzed by Prism 9.0 GraphPad software. For comparison of means between the two groups, the unpaired two-tailed Student’s *t*-test was performed. Statistical comparisons of multiple indicators between two groups were performed using multiple *t*-tests. A *p*-value of <0.01 was considered statistically significant.

## Results

### Case report

**Figure 1.**
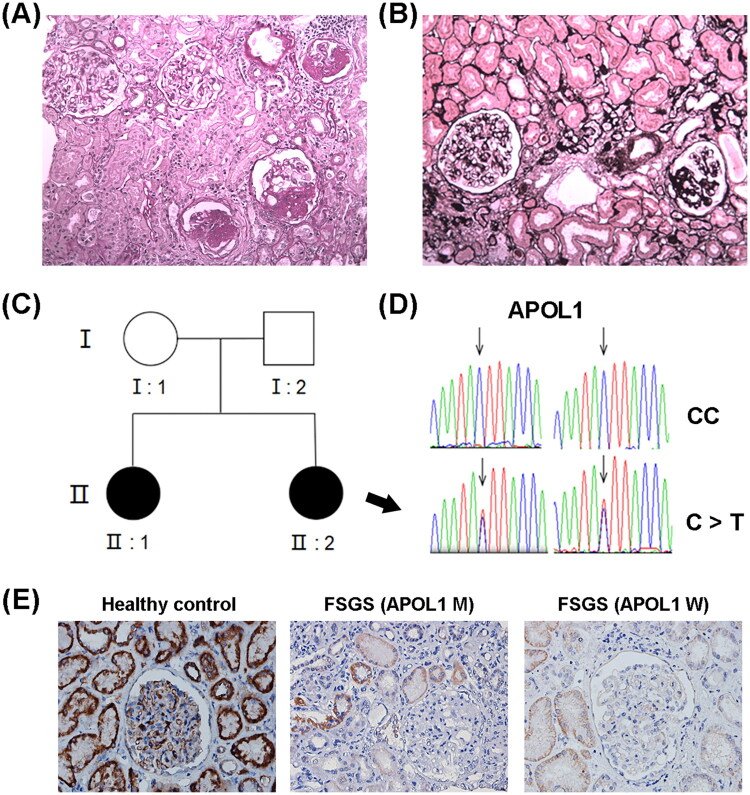
Novel *APOL1* gene mutation in twin patients with FSGS. (A) Periodic Acid-Schiff stain (×100). Four glomerulus exhibited obvious spherical sclerosis, with slight hyperplasia of mesangial cells and matrix in other smaller glomeruli. No significant pathological alterations were observed in the mesangial region and basement membrane. (B) Periodic acid-sliver methenamine stain (×200). Two glomerulus displayed peripheral fibrosis. Renal tubule epithelial cells exhibited denaturation with vacuoles and granules, along with approximately 30% multifocal atrophy and interstitial fibrosis. (C) Pedigree of the FSGS twins. Both twin sisters were diagnosed with FSGS. (D) Heterozygous missense mutation in exon 5 (c.815 C > T, *p.T272I*) identified within the *APOL1* gene. The top sequence represents the wild type sequence; the bottom sequence corresponds to the mutant sequence. (E) Immunohistochemistry (×400) for *APOL1* in renal biopsies. Compared to healthy controls, *APOL1* staining was markedly reduced in both glomeruli and tubules of FSGS patients without *APOL1* mutations (*APOL1*-WT) and those carrying the mutation (*APOL1*-MUT). Notably, glomerular *APOL1* expression was significantly lower in *APOL1*-MUT than in *APOL1*-WT patients.

A 20-year-old girl was admitted to the hospital with a complaint of intermittent edema and proteinuria for 15 days. Laboratory assessments conducted at a local medical facility revealed 24-h proteinuria of 7.02 g, accompanied by hypoalbuminemia (28.7 g/L) and hypercholesterolemia (6.35 mmol/L). The patient’s renal biopsy exhibited typical non-special FSGS features: evident segmental sclerosis with adhesion, multifocal tubular atrophy, and a large amount of inflammatory cell infiltration in the interstitial with mild fibrosis (Figure 1A,B).

### Whole Genome sequencing

Whole genome sequencing was conducted on the FSGS-afflicted patient, her twin sister, as well as their parents. This comprehensive genetic exploration led to the discovery of a previously unreported missense mutation (*c.C815T*, *p.T272I*; RefSeq NM_001136540.2) within the *APOL1* gene in both twin sisters. Notably, this mutation was absent in the parental genomes (Figure 1C,D). To validate the levels of *APOL1* expression in renal tissues of FSGS patients carrying the mutation, we obtained deidentified, formalin-fixed, paraffin-embedded human kidney specimens from the proband, FSGS without *APOL1* mutation, and healthy control (Figure 1E). The results showed significantly reduced *APOL1* expression in FSGS patients versus healthy controls. While both *APOL1* M and *APOL1* W exhibited reduced expression, tubular *APOL1* staining was stronger in *APOL1* M than in *APOL1* W (Figure 1E), indicating mutation-associated differences in renal localization.

**Figure 2.**
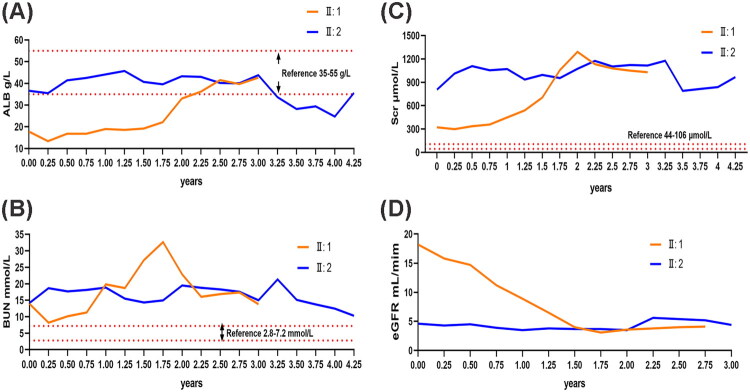
Longitudinal biochemical parameters in FSGS twins. (A) ALB, albumin; (B) BUN, blood urea nitrogen; (C) Scr, serum creatinine; (D) eGFR, estimated glomerular filtration rate.

Remarkably, seven months later, the twin sister was admitted to the hospital due to the presence of edema and proteinuria, ultimately receiving a diagnosis of FSGS. Presently, both patients are undergoing dialysis treatment. Follow-up data are provided in Figure 2.

### APOL1 protein structure and the prediction of the mutation structure

**Figure 3.**
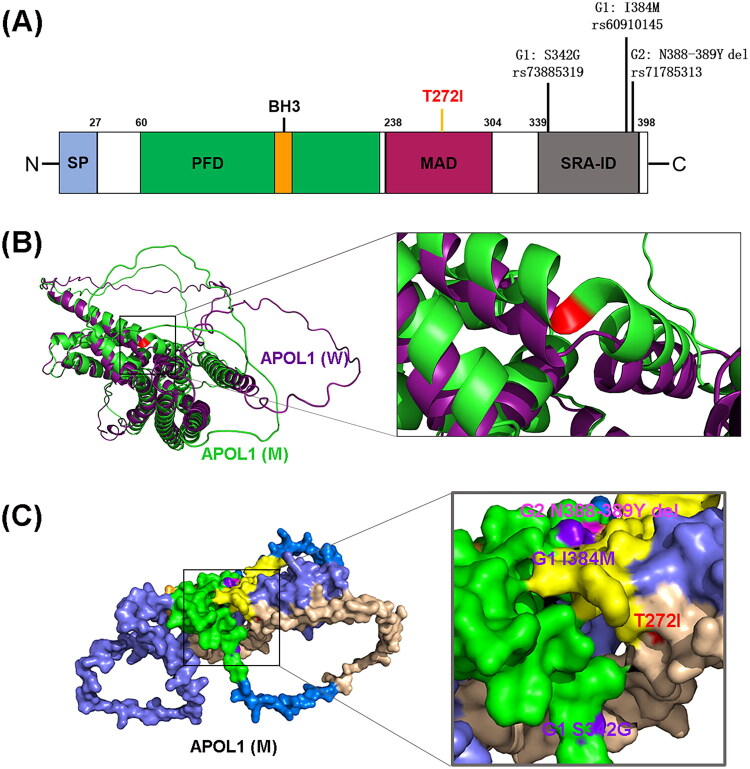
Schematic structure and three-dimensional modeling of the *APOL1* protein. (A) Domain organization of *APOL1* isoform a: Signal peptide (SP, aa 1–27); bcl-2 homology 3 domain (BH3, aa 158–166); pore-forming domain (PFD, aa 60–235); membrane address domain (MAD, aa 238–304); serum resistance-associated interaction domain (SRA-ID, aa 339–392). *G1/G2* mutations localize to SRA-ID; the novel *p.T272I* variant resides in MAD. (B) Predicted 3D structures for wild-type *APOL1* (*APOL1*-WT) and *p.T272I* mutant (*APOL1*-MUT) based on AlphaFold2 modeling (NM_001136540.2). (C) Spatial location of *G1*, *G2* and *p.T272I* mutations in the *APOL1* 3D structure predicted by AlphaFold2.

The commonly encountered *APOL1* isoform a (NM_001136540.2) has 398 amino acids and a molecular weight of approximately 42 kDa [11,35,36]. This isoform possesses a signal peptide that allows *APOL1* export from specific cell types, and it only exists in *APOL1* protein of APOL family. Its remaining three primary domains are characterized by their functions in trypanolysis [37]. The N-terminal pore-forming domain (PFD) exhibits structural resemblances to bacterial colicins and also contains a BH3-only domain, suggesting a potential involvement in autophagy or apoptosis. The membrane-addressing domain (MAD) may function as a pH-dependent switch, implicated in targeting the trypanosome lysosome for pore formation and altering binding to lipids containing particles. The C-terminal serum resistance-associated (SRA) interaction domain (SRA-ID) is inhibiting the pore-forming activity through direct interaction with SRA protein of *Trypanosoma brucei rhodesiense (T.b. rhodesiense)*. Notably, established *APOL1* risk variants *G1* (*S342G/I384M*) and *G2* (*ΔN388-Y389*) reside within SRA-ID, while our novel *p.T272I* variant localizes to MAD (Figure 3A).

The restricted phylogenetic distribution of *APOL1* implies it is nonessential for baseline renal function. A prevailing consensus in most studies suggests that the prevalent *APOL1* risk alleles, *G1* and *G2*, follow a gain-of-dysfunction mechanism [12]. In the context of the FSGS twins involved in this study, harboring a missense mutation (*p.T272I*) within the *APOL1* gene that resulted in the conversion of the hydrophilic amino acid threonine to the hydrophobic amino acid isoleucine. Since the protein structure of *APOL1* remains undetermined, in order to elucidate the potential impact of this novel mutation on the protein’s structure and function, we performed a three-dimensional (3D) structure prediction of the mutated *APOL1* protein by AlphaFold2 [38]. We overlapped the predicted 3D structures of mutated (*p.T272I*) and wild-type *APOL1* (AF-O14791-F1, https://alphafold.ebi.ac.uk/entry/O14791). As depicted in Figure 3B, the mutant *APOL1* superimposes well with wild-type *APOL1*, affirming the reliability of both models. However, the original coil structure underwent a transformation into a helix subsequent to the amino acid mutation from T to I at position 272, which may enhance the rigidity of *APOL1*. The spatial locations of the two high-risk mutations, *G1* and *G2*, as well as the new mutation of this project (*p.T272I*), in the predicted 3D structure of the *APOL1* protein, are shown in Figure 3C.

**Figure 4.**
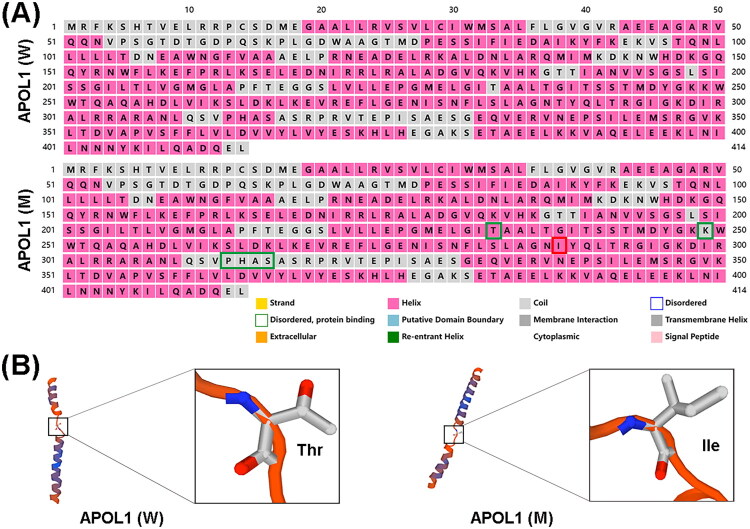
Secondary structure prediction for *APOL1* isoform b (NM_145343.3). (A) Change of secondary structure of the new variant in *APOL1* isoform b (NM_145343.3). Green boxes: Helical conformation changes; red boxes: Amino acid substitution. (B) The backbone atom ribbon structures show the and these inter-helical orientation changes. *APOL1* (W), *APOL1* wild type (G0); *APOL1* (M), *APOL1* mutation (T272I); thr, threonine; ile, isoleucine.

Furthermore, we utilized an online protein structure prediction software (http://bioinf.cs.ucl.ac.uk/psipred/) to assess the influence of the new mutation on *APOL1* isoform b (NM_145343.3, which has a longer N-terminus compared to isoform a). The outcome of the target sequence analysis indicated that the new mutation within *APOL1* corresponds to amino acid position 288 in NM_145343.3 (Figure 4A). The secondary structure map illustrates that the structural models of the N-terminus and C-terminus in both the new variant (*APOL1* (M)) and wild-type (*APOL1* (W)) exhibit comparable lengths of secondary structured elements. However, there is a notable alteration in the helix and coil structural elements surrounding the mutation site in comparison to *APOL1* (W). The details insight of the structural alterations is shown in Figure 4B.

### The novel APOL1 mutation causes impaired morphology and function of podocytes

**Figure 5.**
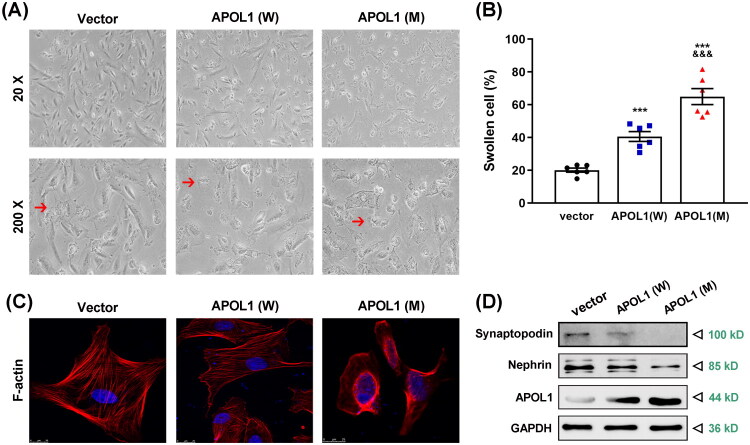
The novel *APOL1* mutation is associated with increased podocyte damage. (A) Morphology of transfected podocytes (48 h; bright-field microscopy). Arrows denote vacuoles in degenerating cells. Scale bars: 20× (top), 200× (bottom). (B) Quantification of swollen cells. The quantitative results (mean ± SEM) are from three sets of experiments; each group takes 6 photos at a time and counts the number of living and dead cells. ***p < 0.001 compared to vector group, and ^&&&^*p* < 0.001 compared to *APOL1* (W) group. (C) Immunofluorescence staining of podocyte cells after transfection with different plasmids for 48 h. F-actin was stained by Alexa Fluor 568 Phalloidin (red), and counterstained with nuclear dye 49,6-diamidino-2-phenylindole (DAPI; blue). Stained cells were observed under immunofluorescence microscopy. Merged images of all elements and colocalized images of the detected markers are presented at original magnification. Representative microphotographs are displayed. (D) Expression of *APOL1*, nephrin, and synaptopodin was analyzed by immunoblotting in podocytes transfected with wild-type and mutant plasmids of the *APOL1* gene. GAPDH served as a loading control. A representative experiment out of at least three is shown. *APOL1* (W): *APOL1* wild-type (G0); *APOL1* (M): *APOL1* mutation (*c.C815T*, *p.T272I*).

To validate the impact of the novel variant on podocyte damage, we introduced the new mutant *APOL1* protein in podocytes through the construction of plasmids. By calculating the ratio of viable within each group, we observed that the cytoplasm of the podocytes in the mutant group (*APOL1* (M)) exhibited substantial lysis and fragmentation, accompanied by an enlargement of intercellular gaps (Figure 5A). Upon quantification, the data demonstrated a significantly higher percentage of cell death in the *APOL1* (M) group compared to both the vector and wild-type groups (*p* < 0.001). This observation indicated that the new *APOL1* mutation contributed to an exacerbation of cell death to some degree (Figure 5B). Notably, immunoblot analysis confirmed that *APOL1* (M) protein expression was higher than that of *APOL1* (W) in transfected cells (data not shown), which may partially account for the increased toxicity. In addition, the proportion of cell death in the wild-type plasmid group (*APOL1* (W)) was higher than that in the vector group (*p* < 0.001), suggesting that *APOL1* overexpression itself might induce podocyte damage. Consistent with the cell death data, F-actin staining appeared more intense in the *APOL1* (M) group compared to *APOL1* (W) (Figure 5C), which may reflect enhanced cytoskeletal remodeling associated with cellular swelling—a phenotype potentially amplified by both the mutation and higher expression levels of *APOL1* (M).

Given podocytes’ terminally differentiated nature and actin-dependent architecture, we assessed cytoskeletal integrity by confocal immunofluorescence. The results showed that in normal podocytes, the cytoskeletal protein F-actin aggregated into bundles of microfilament-like structures, which were distributed along the long axis of the cell to form stress fibers (Figure 5C). However, when podocytes expressed the new mutant protein, F-actin expression was reduced, and microfilament-like structures disintegrated into disorganized and attenuated skeletal proteins, leading to cytoskeletal lysis and an increase in cytosolic interstitial space. These results indirectly reflect the shrinkage of the podocyte foot process. In podocytes expressing wild-type *APOL1* protein, F-actin showed some degree of solubilization, the clarity of backbone proteins was reduced, and their boundaries became blurred. This result indicated that overexpression of *APOL1* was toxic to podocyte cells.

The above results show the impact of the new *APOL1* mutation on podocyte viability and cytoskeleton morphology. Therefore, we proceeded to delve into the effect of this mutation on functional proteins in podocytes. Total podocyte protein was extracted after transfection with the plasmid for 48 h. This assessment encompassed the detection of *APOL1* protein expression, as well as the expressions of nephrin and synaptopodin, which serve as markers for podocyte maturation. The results revealed a significant increase in the levels of *APOL1* protein in both transfected groups. However, the expression levels of synaptopodin and nephrin were markedly reduced in the *APOL1* (M) group (Figure 5D). This outcome indicated that both the *APOL1* mutation protein and the overexpression of wild-type protein have an impact on the normal function of podocytes, with the mutant protein exerting a more significant effect on causing injury to podocytes.

### The novel APOL1 mutation is associated with mitochondrial damage and altered autophagic markers by altering chloride ion (Cl^-^) flow

**Figure 6.**
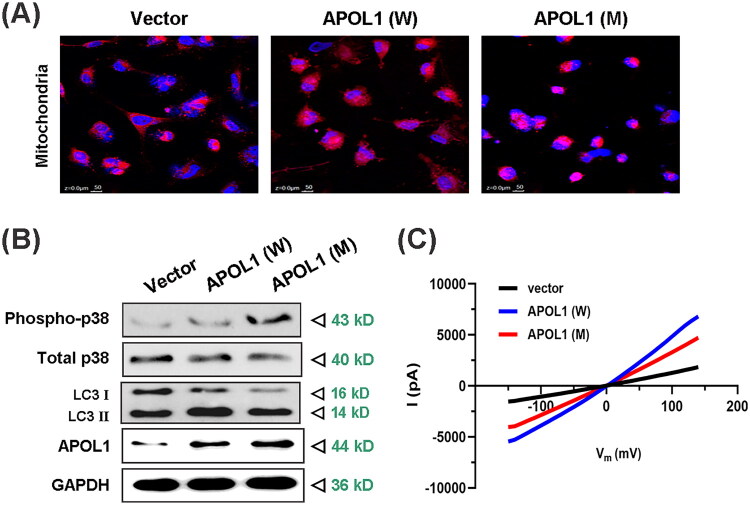
*APOL1 p.T272I* Induces mitochondrial damage, autophagic markers dysregulation, and altered Cl^-^ flux. (A) Immunofluorescence staining of podocyte cells after transfection with different plasmids for 48 h. Mitochondria (red) and counterstained with nuclear dye 4’,6-diamidino-2-phenylindole (DAPI; blue). Stained cells were observed under immunofluorescence microscopy. Merged images of all elements and colocalized images of the detected markers are presented at original magnification. Representative microphotographs are displayed. (B) Expression of *APOL1*, LC3II/LC3I, phospho-*p38*/*p38* was analyzed by immunoblotting in podocytes transfected with wild-type and mutant plasmids of the *APOL1* gene. GAPDH served as a loading control. A representative experiment out of at least three is shown. (C) Whole-cell membrane currents were recorded in HEK293 cells after transfected with plasmids for 48 h. Representative current-voltage relationships (I-V) of the cell obtained in bath solution of the indicated pH7.4 (*n* = 3 replicates, 3–5 cells/group). *APOL1* (W): *APOL1* wild-type (G0); *APOL1* (M): *APOL1* mutation (*c.C815T*, *p.T272I*).

**Figure 7.**
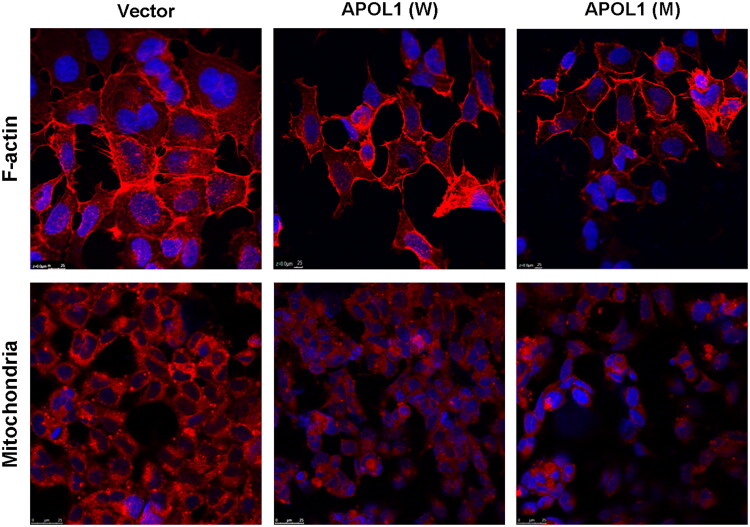
Cytoskeletal and mitochondrial alterations in HEK293 cells. Immunofluorescence staining of HEK293 cells 48 h after transfection with different plasmids. Stained cells were observed under immunofluorescence microscopy: F-actin (red), mitochondria (red) and nuclear (blue). Merged images of all elements and colocalized images of the detected markers are presented at original magnification. Representative microphotographs are displayed. *APOL1* (W), *APOL1* wild type (G0); *APOL1* (M), *APOL1* mutation (T272I).

*APOL1* risk variants *G1/G2* reportedly induce cell death through mitochondrial dysfunction [39]. We therefore assessed mitochondrial integrity in podocytes expressing the novel T272I variant (Figure 6A). The results unveiled that both the expression of *APOL1* new mutant protein and the overexpression of *APOL1* wild-type protein induced severe impairment to mitochondria. In addition, we replicated these results in human embryonic kidney (HEK) 293 cells (Figure 7), and the results showed that the effect of *APOL1* mutant protein on HEK293 cells was consistent with that in podocytes.

Given that autophagy and *p38* activation are recognized as potential hallmarks of cytotoxicity in podocytes harboring *APOL1* risk mutations [40], we also investigated the influence of this novel *APOL1* mutation on the regulation of the *p38* and autophagy signaling pathways in podocytes. Phospho-*p38* levels increased significantly in transfected podocytes (Figure 4B), indicating *p38* pathway activation. The LC3-II/LC3-I ratio also increased in both *APOL1*-MUT and *APOL1*-WT groups [41], suggesting autophagic marker dysregulation (functional flux requires future validation). These findings suggested that the novel *APOL1* mutation may incite podocyte damage by activating the stress-activated protein kinases *p38*. Moreover, autophagy, recognized as an authentic stress-responsive mechanism, is also spurred into action in response to the podocyte toxicity triggered by the new *APOL1* mutant protein. The toxic effects observed due to overexpression of the wild-type *APOL1* protein in podocytes align with the results reported in related studies [36].

The death-promoting activity of *APOL1* has been reported to be associated with the potential for ion transport across intracellular membranes, and the loss of cellular ion gradients could lead to osmotic swelling and eventually cell death. *APOL1*, in fact, imparts defense against protozoan parasites by forming channels within the pathogens, leading to ion flux that drives the process of trypanolysis [37]. However, there has been controversy surrounding the ion selectivity of *APOL1*. Initial reports pointed toward Cl^-^ selectivity [8], and recent research provided evidence that *APOL1* channels conduct Cl^-^ at low pH and K^+^ when the pH is neutralized [42]. Nonetheless, studies suggest that *G1* and *G2* risk variants exhibit heightened cytotoxicity under acidic conditions due to altered pH sensitivity, with evidence pointing to enhanced cation channel activity under specific pH environments [25,43]. The two risk mutations, *G1* and *G2*, are not present in the Chinese population. In addition, the PFD of *APOL1* has been involved in anion channel formation and cell death in trypanosomes, and secondary structure predictions implied that the novel mutation could impact the structure of *APOL1*’s PFD. Considering in light of the above, we investigated the effect of the new mutation and wild-type *APOL1* protein on cellular chloride channels using the membrane clamp technique. Since the extended period required for podocyte differentiation to mature, we employed HEK293 cells transfected with different plasmids to detect alterations in ion channels, calculating the current values at different time intervals. Consistent with *APOL1*’s established role in ion flux [1], both wild-type (WT) and mutant (T272I) *APOL1* significantly enhanced chloride currents versus vector control. However, the T272I mutant exhibited markedly attenuated chloride conductance, yielding a distinct hierarchy: Vector < T272I < WT (Figure 6C). This intermediate phenotype indicates the mutation partially impairs—but does not abolish—*APOL1*-mediated chloride permeability. We note HEK293 cells were used due to technical constraints in maintaining differentiated podocytes for extended patch-clamp studies; while informative, this model may not fully recapitulate podocyte-specific channel biology [44].

## Discussion

Apolipoprotein L1 (*APOL1*) is a trypanolytic pore-forming protein in blood, conferring natural resistance to most African trypanosome infections in humans. The *Trypanosoma brucei* subspecies *rhodesiense* has evolved to counteract the lytic action of *APOL1* G0 by producing an SRA protein, which interacts with the C-terminal helix of *APOL1* [45]. The amino acid substitution is caused by two functional mutations (*G1: rs73885319 A > G*, and *rs60910145 T > G*; *G2: rs71785313 TTATAA/-*) is responsible for *APOL1* evasion of SRA binding. Structural modeling hints that these variants might perturb coiled-coil interactions with SRA in the C-terminal protein domain, thus allowing *APOL1*-directed trypanolytic to proceed unimpeded [46]. As a consequence of natural selection, the frequency of *APOL1* risk variants is considerably higher in individuals of sub-Saharan African ancestry. The impact of *APOL1* on innate immunity and kidney disease has undergone extensive exploration since its initial discovery by Duchateau et al. in 2001 [13,47,48]. Numerous investigations have demonstrated a connection between the two risk variants and an elevated susceptibility to various kidney disorders, along with an augmented risk of progressing to ESRD, particularly among individuals of AAs descent. Such conditions include FSGS, hypertensive nephropathy (HTN), human immunodeficiency virus-associated nephropathy (HIVAN), etc. [49,50].

However, the role of *APOL1* risk variants in populations beyond African Americans (AAs) has received limited attention in previous studies. Before initiating this study, we conducted an analysis of genetic variations and haplotype diversity with *APOL1* gene using the 1000 Genomes database [51], along with an assessment of *APOL1* gene copy number variation (CNV) in Chinese FSGS patients [52]. The findings unveiled notable disparities in the distribution of variants and haplotypes within both the regulatory and coding regions of the *APOL1* gene between Asian populations and others. However, no significant correlation was observed between *APOL1* gene CNVs and either FSGS susceptibility or clinical manifestations in the Chinese population. These findings suggest significant diversity in *APOL1* gene variants among different populations. Beyond the well-studied *G1* and *G2* variants, there may be other, yet undiscovered variants that could be associated with kidney diseases prevalent in Asian populations. In this study, we identified a novel *APOL1* mutation (*c.C815T*, *p.T272I*) in Chinese patients with focal segmental glomerulosclerosis (FSGS). Notably, this mutation was detected in twin sisters diagnosed with FSGS but was absent in their unaffected parents, strongly suggesting its potential pathogenic role. According to the ACMG/AMP guidelines [53–55], the *p.T272I* variant is classified as Pathogenic. This classification is supported by compelling evidence: whole-genome sequencing confirmed the variant’s *de novo* origin and its presence in both affected twins, demonstrating complete cosegregation with the FSGS phenotype (PS2). Furthermore, the twins exhibited clinical features highly characteristic of *APOL1*-associated nephropathy, including nephrotic-range proteinuria (7.02 g/24 h), hypoalbuminemia, hypercholesterolemia, biopsy-confirmed FSGS, and progression to dialysis, providing strong phenotypic support for its pathogenicity (PP1/PM1). Critically, the *p.T272I* variant was absent from major population databases such as the Genome Aggregation Database (gnomAD) (https://gnomad.broadinstitute.org/), the 1000 Genomes Project (www.internationalgenome.org), The Exome Aggregation Consortium (ExAC) (https://ngdc.cncb.ac.cn/databasecommons/database/), Chinamap (www.mbiobank.com/), NARD (https://nard.macrogen.com/) and Bio-Med Big Data Center (https://www.biosino.org/wepd) [56–62], confirming its rarity consistent with pathogenic variants in Mendelian disorders (PM2). While future population-scale studies will refine penetrance estimates of the *p.T272I* variant, our ACMG/AMP classification (Pathogenic; PS2, PP1/PM1, PM2) and comprehensive database interrogation confirm both its extreme rarity and causal role in this familial context.

This base missense alteration resulted in the replacement of the hydrophilic threonine at position aa272 in the MAD of *APOL1* with a hydrophobic isoleucine. Structural modeling outcomes revealed an exchange between α-helix and coil structures in both the PFD and MAD of the new mutant *APOL1* protein (Figure 3B). The MAD of *APOL1* is necessary for association with the cell membrane and is also required for *APOL1* toxicity [11]. Additionally, MAD is reported to consist of pH-sensitive a-helices that act as a pH-dependent switch and facilitate the association with HDL particles at a neutral pH or intracellular regions at an acidic pH [36,63]. According to the putative working model for *APOL1* topology on podocytes and in serum reported by Gupta et al. *APOL1* is localized on the surface of podocytes, with most of the PFD and C terminus of the SRA-ID, but not the MAD, being exposed. In contrast, differential trypanolytic blocking activity reveals that the MAD is exposed in serum. These findings highlighted different conformations of native *APOL1* topology in serum (HDL particles) and at the podocyte surface, thereby lending support to the surface ion channel model for *APOL1* risk variant–mediated podocyte injury [64]. A frameshift mutation located between the two alpha α-helixes of the MAD has been documented to result in an Indian individual’s infection with *T. evansi*. This mutant is unable to trigger trypanolysis due to the requisite presence of both intact PFD and MAD for trypanosome-killing activity [65]. However, it is noteworthy that the Indian individual bearing this MAD mutation in *APOL1* has not been reported to suffer from kidney-related diseases. This observation implies that different mutation sites cause alterations in *APOL1* can lead to alterations in its function, with varying degrees of risk associated with different types of diseases.

The MAD of *APOL1* is assumed to mediate membrane targeting together with the PFD [37,66]. In podocytes, it is likely that the MAD is located extracellular and is inaccessible due to being blocked by some hypothetical proteins. The identified T272I mutant *APOL1* protein lacks hydrophilic amino acids, possibly leading to the disruption of hydrogen bonds, helicity, and conformational stability around the mutation site. Reports suggest that *APOL1* cytotoxicity could result from mis-localization of the protein [67,68]. Thus, the increase in hydrophobicity caused by the mutation may alter MAD’s ability to bind to other proteins, subsequently affecting *APOL1*’s localization on the podocyte membrane and causing cytotoxicity. This speculation is supported by our findings regarding the viability of podocytes expressing the mutant protein (Figure 5A). Additionally, as compared to wild-type *APOL1* protein, the increase of helix at the aa272 mutation site in the 3D structure prediction model may alter the packing of adjacent helices and the conformational flexibility of the *APOL1* variant. Thus, this newly identified mutation could significantly affect *APOL1*’s function, thereby increasing carriers’ susceptibility to FSGS. Furthermore, *APOL1* has been shown to regulate PI(4)P synthesis through MAD interactions, thereby influencing mitochondrial fission and autophagy *via* PI4KB activity [69,70]. Our results demonstrated that the T272I mutation in MAD of *APOL1* is a potent inducer of mitochondrial dysfunction and accumulation of LC3-II (Figures 6A,B and 7), leading to vacuole formation and podocyte death (Figure 5A). Consistent with our findings, Wan et al. established *APOL1* as an inducer of autophagic cell death, which triggers time-dependent accumulation of lipidated LC3-II, autophagosome formation, and subsequent cell death [71]. Critically, *APOL1*-mediated lysosomal dysfunction and impaired autophagic flux may directly or indirectly exacerbate mitochondrial damage [72,73]. The observed LC3-II accumulation coupled with mitochondrial dysfunction thus supports a model wherein dysregulation of autophagic markers, in conjunction with *APOL1* variants, contributes to FSGS pathogenesis [74,75]. Future studies will examine autophagic flux dynamics to further validate this mechanism. Notably, cytoskeletal integrity in podocytes is critically dependent on ion homeostasis and mitochondrial function. Disrupted chloride gradients—as observed in *p.T272I*-expressing cells—may impair actin dynamics through dysregulation of chloride-sensitive cytoskeletal regulators (e.g., gelsolin family proteins), potentially contributing to foot process effacement in FSGS [22,23,76]. As a result, the novel *APOL1* variant could potentially exert pathophysiological effects on renal disease by affecting the podocytes’ function. The IHC results demonstrated a dramatically decreased in *APOL1* expression levels in FSGS patients compared with healthy control, and even lower levels were observed in glomeruli of FSGS patients with *APOL1* mutation. This finding aligns with the report by Sethu M Madhavan [77]. Consequently, it can be inferred that the lysis of podocytes in glomeruli may contribute to the loss of *APOL1* protein.

The cytotoxic mechanism of *APOL1* is intrinsically linked to dysregulated ion transport across intracellular membranes. While wild-type *APOL1* (G0) lacks pathogenic ion channel activity, established risk variants (*G1/G2*) drive cell death through altered cation flux (K^+^/Na^+^/Ca^2+^) [19–21,24,40,43,78]. Our electrophysiological data reveal that the novel *p.T272I* mutant exhibits residual chloride influx compared to vector controls, though with significantly attenuated conductance relative to G0 (Figure 6C). Critically, this reduction does not reflect altered ion selectivity: *p.T272I* maintains anion permeability without acquiring pathogenic cation conductance (no elevated K^+^/Na^+^ flux) [11,48,79]. This fundamentally distinguishes it from *G1/G2* variants, which gain pH-sensitive cation channel activity under acidic conditions [20,21,40]. Mechanistically, *p.T272I* selectively impairs anion transport, contrasting with the enhanced cation conductance of *G1/G2*. Although *G1/G2* variants show pronounced activation in acidic environments—a property not yet assessed for *p.T272I*—this warrants investigation given *APOL1*’s known pH-dependent ion switching [24,42]. Pathogenically, whereas *G1/G2* drive rapid podocyte necrosis *via* cation-mediated osmotic shock and mitochondrial dysfunction [20,21,25], *p.T272I*’s chloride dysregulation likely induces milder autophagic stress through disrupted electrochemical gradients. This aligns with its intermediate cytotoxicity phenotype (Vector < Mutant < Wild-type). Biologically, the consequences of chloride-specific toxicity diverge from cation-mediated damage. We therefore classify *p.T272I* as a partial loss-of-function variant relative to G0, distinct from the gain-of-toxic-function mechanism of *G1/G2*, due to its diminished yet persistent ion transport capacity. Therapeutically, Inaxaplin (an *APOL1* cation channel blocker [80,81]) may still benefit *p.T272I* carriers if secondary cation imbalances occur—a testable hypothesis for future studies.

While our study identifies a novel *APOL1* variant associated with FSGS and provides functional evidence for its pathogenicity, several limitations warrant acknowledgment: The absence of extended familial segregation precludes definitive confirmation of its *de novo* origin; lack of systematic screening for podocytopathy genes (*NPHS1*, *NPHS2*, *INF2*, etc.) prevents exclusion of alternative genetic contributors. Functionally, potential overexpression artifacts from supraphysiological expression and absence of rescue experiments limit mechanistic certainty, while incomplete quantitative validation (e.g., mitochondrial OCR/ΔΨm/ROS) constrains characterization of cellular dysfunction and the need for future authophagic flux-based validation. Modeling limitations include modest assay sample sizes (despite substantial effect sizes) and unvalidated computational predictions of structural impacts. Finally, the observed dominant effect in heterozygotes contrasts with biallelic *G1/G2* mechanisms, leaving potential contributions from genetic modifiers or incomplete penetrance unresolved.

## Data Availability

All original data supporting the results reported in the article are available upon request.
